# Methodological Appraisal of Literature Concerning the Analysis of Genetic Variants or Protein Levels of Complement Components on Susceptibility to Infection by Trypanosomatids: A Systematic Review

**DOI:** 10.3389/fimmu.2021.780810

**Published:** 2021-11-25

**Authors:** Thais Cristina Tirado, Larine Lowry Moura, Patrícia Shigunov, Fabiano Borges Figueiredo

**Affiliations:** ^1^ Laboratório de Biologia Celular, Instituto Carlos Chagas, Fundação Oswaldo Cruz (FIOCRUZ), Curitiba, Brazil; ^2^ Laboratório de Biologia Básica de Células-Tronco, Instituto Carlos Chagas, Fundação Oswaldo Cruz (FIOCRUZ), Curitiba, Brazil

**Keywords:** complement system, polymorphism, expression levels, susceptibility, leishmaniasis, Chagas Disease, trypanosomatids, systematic review

## Abstract

**Background:**

Trypanosomatids are protozoa responsible for a wide range of diseases, with emphasis on Chagas Disease (CD) and Leishmaniasis, which are in the list of most relevant Neglected Tropical Diseases (NTD) according to World Health Organization (WHO). During the infectious process, immune system is immediately activated, and parasites can invade nucleated cells through a broad diversity of receptors. The complement system − through classical, alternative and lectin pathways − plays a role in the first line of defense against these pathogens, acting in opsonization, phagocytosis and lysis of parasites. Genetic modifications in complement genes, such as Single Nucleotide Polymorphisms (SNPs), can influence host susceptibility to these parasites and modulate protein expression.

**Methods:**

In March and April 2021, a literature search was conducted at the PubMed and Google Scholar databases and the reference lists obtained were verified. After applying the inclusion and exclusion criteria, the selected studies were evaluated and scored according to eleven established criteria regarding their thematic approach and design, aiming at the good quality of publications.

**Results:**

Twelve papers were included in this systematic review: seven investigating CD and five focusing on Leishmaniasis. Most articles presented gene and protein approaches, careful determination of experimental groups, and adequate choice of experimental techniques, although several of them were not up-to-date. Ten studies explored the association of polymorphisms and haplotypes with disease progression, with emphasis on lectin complement pathway genes. Decreased and increased patient serum protein levels were associated with susceptibility to CD and Visceral Leishmaniasis, respectively.

**Conclusion:**

This systematic review shows the influence of genetic alterations in complement genes on the progression of several infectious diseases, with a focus on conditions caused by trypanosomatids, and contributes suggestions and evidence to improve experimental design in future research proposals.

## 1 Introduction

Trypanosomatids are flagellated protozoan parasites present in tropical and subtropical areas that cause a wide range of diseases in humans and animals, such as Chagas Disease (CD), Cutaneous and Visceral Leishmaniasis (CL and VL, respectively), and African sleeping sickness (1), which are mainly transmitted by vector insects (2). Most of the diseases caused by trypanosomatids can result in morbidity and mortality in adults, children, and animals. About half a billion people live in areas at risk for these diseases, and more than 20 million have infections caused by at least one of them, accounting for over 100,000 deaths a year (1, 3).

Leishmaniasis and CD are in the list of most relevant Neglected Tropical Diseases (NTD) according to the World Health Organization (WHO) caused by infection with *Leishmania* spp. and *Trypanosoma cruzi*, respectively (4). CD, which is transmitted by triatomine bugs, is endemic in Latin America (5, 6). CL and VL, on the other hand, are transmitted by infected phlebotomine sandflies mainly in poor and underprivileged populations (7), and are considered the two main distinct manifestations of Leishmaniasis (8). VL, the most severe form, is caused by *Leishmania infantum* and *Leishmania donovani* in the Americas and Eastern Hemisphere, respectively. It affects people every year and can present high death rates if not properly treated (9). CL is caused by many *Leishmania* species of subgenus *Leishmania* and *Viannia* in Africa, Asia, and Latin America (9, 10).

During the infection process, the parasites can invade several nucleated cells of the host through a broad variety of receptors (11). One of the first defense mechanisms of the hosts is complement activation, which recognizes and controls parasite invasion and presents important biological responses such as opsonization, phagocytosis and lysis of microorganisms and cells (12, 13), acting through classical, alternative and lectin pathways (12). The classical pathway is initiated by immune complexes, depending mainly on the interaction of C1 with antigen-antibody complexes, or occasionally by pathogen-associated molecular patterns (PAMPs) (12, 14, 15). The lectin pathway starts the activation process, and is triggered by the binding of pattern-recognition molecules (PRMs), such as mannose-binding lectin (MBL), ficolins and collectins, to PAMPs on parasite surface (15–17). It prompts the activation of the alternative pathway, which occurs when the bond between the C3 α-chain and thiol-ester hydrolyzes spontaneously, exhibiting a reactive site (12). Nevertheless, trypanosomatids have mechanisms to break recognition by the host immune system and escape complement attack; therefore, the infection depends on parasite persistence and host immune response (15, 18).

Capacity of the parasites to invade the host cells and proceed with the infection process depends on their ability to escape the host protection mechanisms and its genetic background (12, 15). Consequently, genetic modifications to the components of the three complement pathways, such as single nucleotide polymorphisms (SNPs), can have an impact on immunocompetence and influence susceptibility to infectious diseases (19, 20). In this context, human complement receptor 1 (CR1) levels were shown to be modulated by genetic variants in exon 29, which led to decreased expression in CD patients, indicating that low levels of this protein may influence *T. cruzi* infection (21, 22). Furthermore, previous studies have shown that gene polymorphisms are associated with mannose-binding lectin-associated serine protease 2 (MASP-2) and MBL expression levels, and are able to cause immunodeficiency, promote infection progression, and influence several infections in VL (23–25), since those genes are related to the lectin pathway activation (26). An illustration of common *MBL2* polymorphisms are mutations in exon 1, which may lead to translational modifications and diminish serum protein levels (27–29). Still referring to the lectin pathway, ficolin-2 (FCN2) is known to be a highly polymorphic encoding gene, and elevated serum levels may facilitate the development of VL (30–32). Moreover, *FCN2* polymorphisms can also influence the development of the cardiodigestive clinical form in chronic CD (20), delaying complement activation when the genetic modifications interfere with the binding capacity of the variant pathogen molecules toward their ligands (30, 33).

Additionally, studies in this area are scarce, and the reason why some individuals develop diseases more easily than others is still not fully understood. Therefore, this systematic review aims to report polymorphisms and expression levels of complement genes and understand how they are involved in the susceptibility or resistance to infectious diseases caused by trypanosomatids.

## 2 Methodology

### 2.1 Selection of Studies

The methodological structure used in this systematic review was adapted from Vasconcelos et al., 2019 (34).

In March and April of 2021, a literature search was conducted at the PubMed and Google Scholar databases. The obtained reference lists were verified for a search of additional papers matching this review topic. The index terms and inclusion and exclusion criteria applied to the selection of the studies are shown in **Chart 1**.

**Chart 1:** Index terms used in the literature search conducted at the PubMed and Google Scholar databases (top) and inclusion and exclusion criteria applied to select the studies (bottom).

Index Terms	
**PubMed** (((polymorphism) OR (haplotype) OR (genotype) OR (genetic variants) OR (mutation) OR (levels)) AND ((leishmania) OR (trypanosoma) OR (leishmaniasis) OR (chagas disease)) AND (complement)) AND (susceptibility))	**Google Scholar** (polymorphism or haplotype or genotype or “genetic variants” or mutation or levels) (complement) (susceptibility) (leishmania or trypanosoma)
**Applied Criteria**	
**Inclusion** Studies that evaluated genetic variations and/or expression levels in complement system genes in conditions caused by trypanosomatids.	**Exclusion** Studies addressing conditions not caused by trypanosomatids, not associated with complement genes and/or with humans, as well as dissertations or thesis, review articles, book chapters, and letters to editor.

### 2.2 Conditions to Assess the Studies

The selected studies were evaluated according to the criteria defined in **Chart 2** in order to better analyze the quality of publications and define potential limitations of each paper. Articles that were in full agreement with each topic received a score of 1, those with partial agreement received a score of 0.5, and those in full disagreement received a score of 0.

**Chart 2:** Conditions used to evaluate the studies included in this review.

Study on the genetic variants and expression levels of complement genes in conditions caused by trypanosomatids; Assessment of Hardy-Weinberg Equilibrium (HWE) in the sample; Analysis of Linkage Disequilibrium (LD) in SNPs; Evaluation of more than one complement pathway; Evaluation of the entire CoDing Sequence (CDS) region and gene regulation; Randomization and inclusion of gender and ethnicity diversity in experimental groups; Sample size determination; Demonstration of suitable diagnosis methodology; Sample suitability for testing; Use of appropriate control groups; Application of adequate and up-to-date techniques.									

## 3 Results

### 3.1 Main Characteristics of the Articles

**Figure 1:**
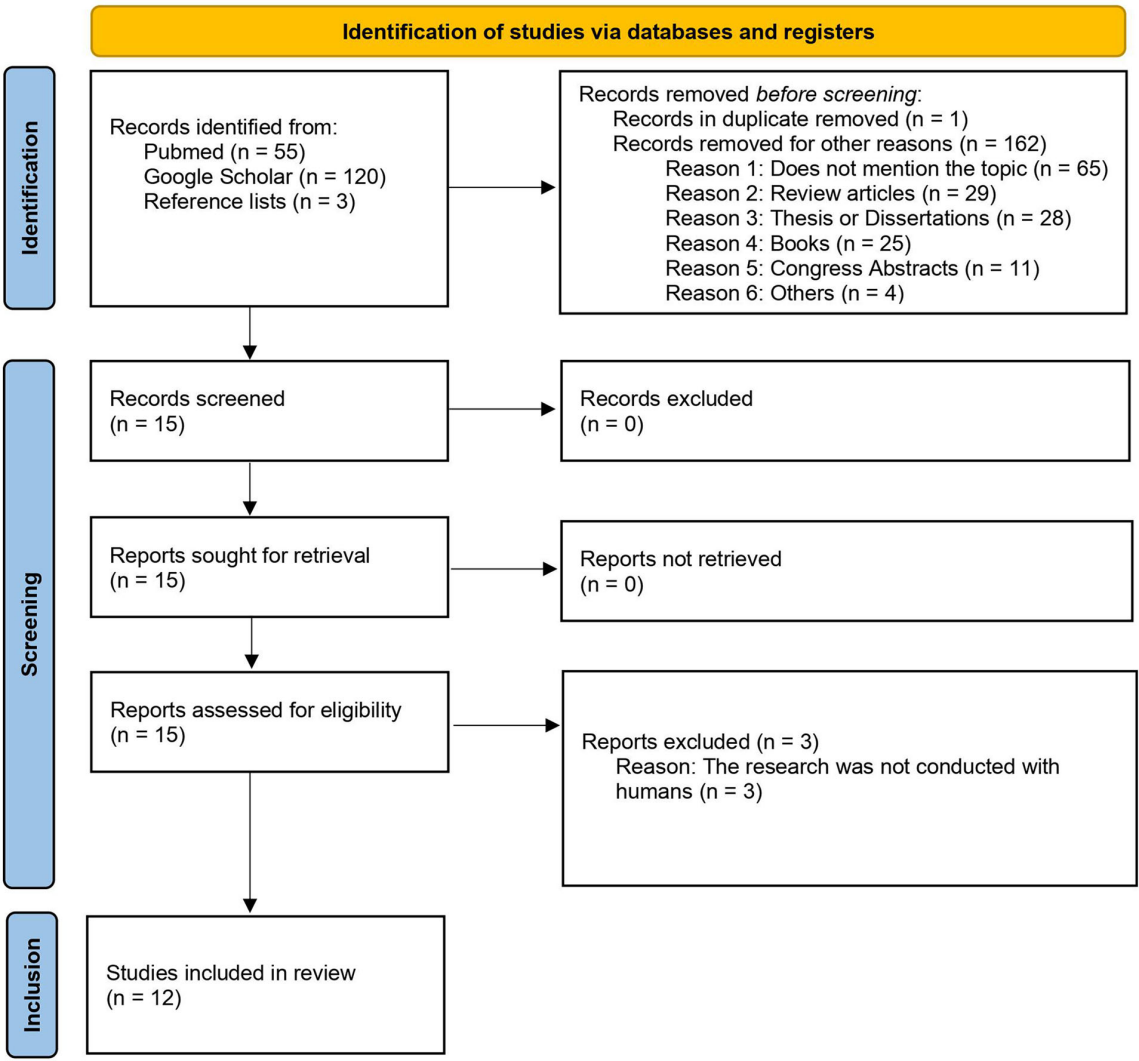
Flowchart summarizing the process used to select the papers for this systematic review in three steps: identification, screening, and inclusion.

A search conducted at the PubMed and Google Scholar databases, with application of the index terms presented in **Chart 1**, retrieved 55 and 120 papers, respectively. The reference lists of all these papers were verified and three more papers were included, totaling 178 studies. One paper, which was found in duplicate in both databases, was removed. After applying the exclusion criteria (**Chart 1**), only 12 studies were included in this systematic review. The selection process was compiled in a flowchart, constructed using the PRISMA tool (35), containing the following sections: identification, screening, and inclusion (**Figure 1**).

**Table 1** shows a summary of the main characteristics of the selected studies.

**Table 1:** Main characteristics of the studies included in this systematic review upon topic, target, methodology and conclusion aspects.

Reference	Main topic	Disease	Location	Control sample size	Patient sample size	Genes	Target*	Genetic variants analysis tools*	Expression levels analysis tools*	Conclusions
Mishra et al., 2015 (32)	Genetic variants and expression levels	Visceral Leishmaniasis (VL)	India	225	218	*FCN2*	Promoter; Exon 8	Sequencing (Sanger)	ELISA	Genetic variants were associated with VL patients. Expression levels increased in VL patients.
Mishra et al., 2015 (36)	Genetic variants and expression levels	Visceral Leishmaniasis (VL)	India	215	218	*MBL2*	Promoter	Sequencing (Sanger)	ELISA	*MBL2* functional variants were associated with VL; Expression levels increased in VL patients.
Sandri et al., 2019 (18)	Genetic variants and expression levels	Chagas Disease (CD)	Brazil	108	251	*COLEC11*	Exon 7	Sequencing (Sanger)	ELISA	Genetic variants were associated with CD patients; Expression levels decreased in CD patients.
Assaf et al., 2012 (8)	Genetic variants and expression levels	Cutaneous Leishmaniasis (CL)	Syria	286	226	*FCN2*	Promoter; Exon 8	Promoter: TaqMan Real-Time PCR Exon 8: Sequencing (Sanger)	ELISA	Genetic variants were associated with CL patients; Expression levels vary among the genetic variants.
Messias-Reason et al., 2003 (37)	Genetic variants (allotypes)	Chagas Disease (CD)	Brazil	100	100	*BF; C3*	*BF S*; *C3 F*	HVAGE	NA	*C3 F* is a susceptible marker for the progression of the cardiomyopathic form; *BF S* may represent a protective role against severe cardiomyopathic disease.
Boldt et al., 2011 (25)	Genetic variants and expression levels	Chagas Disease (CD)	Brazil	300	208	*MASP2*	Exons 3, 8, 10, 12	PCR-SSPs	ELISA	Genetic variants were associated with CD patients; Expression levels decreased in CD patients.
Luz et al., 2016 (38)	Genetic variants	Chagas Disease (CD)	Brazil	202	196	*MBL2*	Promoter; Exon 1	Sequencing (Sanger)	NA	Genetic variants may be associated with CD patients or not.
Sandri et al., 2018 (21)	Genetic variants and expression levels	Chagas Disease (CD)	Brazil	104	232	*CR1*	Exon 29	Sequencing (Sanger)	ELISA	Genetic variants were associated with CD patients; Expression levels decreased in CD patients.
Luz et al., 2013 (20)	Genetic variants and expression levels	Chagas Disease (CD)	Brazil	305	243	*FCN2*	Promoter; Exon 8	Patients: FRET based Real-Time PCR; PCR-SSPs Healthy controls: Sequencing	ELISA	Patients presented lower FCN2 plasma levels than controls; individuals with moderate forms had higher FCN2 levels than the severe forms.
Alonso et al., 2007 (39)	Genetic variants and expression levels	Visceral Leishmaniasis (VL)	Brazil	76	159 (69 = symptomatic; 90 = asymptomatic)	*MBL2*	Promoter; Exon 1	SNaPshot Multiplex kit	Double-antibody immune assay	Genetic variants were associated with VL patients; Expression levels increased in VL patients.
Weitzel et al., 2012 (40)	Genetic variants	Chagas Disease (CD)	Chile	45	125 (64 = symptomatic; 61 = asymptomatic)	*MBL2*	Promoter; MBL2*B; MBL2*C; MBL2*D	RFLP	NA	Low-producer *MBL*2*B genotypes are more common in CD patients.
Asgharzadeh *et al.*, 2007 (41)	Genetic variants	Visceral Leishmaniasis (VL)	Iran	120	58	*MBL2*	MBL2*A (WT)	PCR-SSPs	NA	High-producer MBL2*A genotypes are more common in VL patients.

^*^WT, Wild-Type; PCR, Polymerase Chain Reaction; SSP, Sequence-Specific Primers; FRET, Fluorescence Resonance Energy Transfer; HVAGE, High-Voltage Agarose Gel Elecrophoresis; RFLP, Restriction Fragment Length Polymorphism; ELISA, Enzyme-Linked Immunosorbent Assay; NA, Not Applicable.

Distribution of the papers according to country of origin was as follows: Brazil (7), India (2), Syria (1), Chile (1), and Iran (1). Seven studies focused on CD and five investigated Leishmaniasis in its different manifestations. The score of each paper, according to the established criteria presented in **Chart 2**, is shown in **Chart 3**. These data show that nine papers included analysis of both genetic variants and expression levels. Only two papers did not include the assessment of HWE in its population. LD, defined by the nonrandom association of alleles at different loci, is a sensitive indicator of the population genetic forces that structure a genome (42), and was considered in seven papers. Only one paper evaluated more than one complement pathway. Most of the studies assessed the pathways individually, and the lectin pathway genes/proteins were the target of ten papers. Nine papers included the promoter regulatory region of the gene in polymorphism analysis. Only one paper detailed the randomization scheme of group selection together with the inclusion of gender and ethnicity diversity. In addition, nine papers did not include sample size determination in their experimental designs. No papers considered molecular techniques for disease diagnosis, and most studies used serological techniques. Only one paper did not use an adequate sample for polymorphism analysis. Eight papers were careful about the correct determination of control groups, and no papers included up-to-date or robust techniques for gene and protein analysis.

**Chart 3:** Evaluation of the studies included in this systematic review.

Reference	Criterion number											Final Score (_/11)
	1	2	3	4	5	6	7	8	9	10	11	
Mishra et al., 2015 (32)	1	1	1	0.5	1	0.5	1	0.5	1	1	0.5	9
Mishra et al., 2015 (36)	1	1	1	0.5	1	0.5	1	0.5	1	1	0.5	9
Sandri et al., 2019 (18)	1	1	1	0.5	0.5	0.5	1	0.5	1	1	0.5	8.5
Assaf et al., 2012 (8)	1	1	1	0.5	1	0.5	0	0.5	1	1	0.5	8
Messias-Reason et al., 2003 (37)	0.5	1	NA	1	NA	0.5	0	0.5	1	1	0.5	8
Boldt et al., 2011 (25)	1	1	1	0.5	1	0.5	0	0.5	1	0.5	0.5	7.5
Luz et al., 2016 (38)	1	1	1	0.5	1	0.5	0	0.5	1	0.5	0.5	7.5
Sandri et al., 2018 (21)	1	1	1	0.5	0.5	0.5	0	0.5	1	1	0.5	7.5
Luz et al., 2013 (20)	1	1	0	0.5	1	0.5	0	0.5	1	0.5	0.5	6.5
Alonso et al., 2007 (39)	1	0	0	0.5	1	1	0	0.5	0.5	1	0.5	6
Weitzel et al., 2012 (40)	0.5	1	0	0.5	1	0.5	0	0.5	1	0	0.5	5.5
Asgharzadeh et al., 2007 (41)	0.5	0	0	0.5	1	0	0	0.5	1	1	0.5	5

Captions: 1 – 11: criterion number according to **Chart 2** and as described in the Methodology section. Scores: 0 = study in full disagreement with the criteria; 0.5 = study in partial agreement with the criteria; 1 = study in full agreement with the criteria; NA, not applicable (score = 1).

### 3.2 Summary of Information

#### 3.2.1 Studies Addressing Genetic Variants

Sandri and collaborators (22) investigated the association of six SNPs from the *CR1* exon 29 gene with CD progression. Those authors performed comparisons considering different chronic manifestations. In an experiment conducted with 102 controls and 220 CD patients, they demonstrated that the rs17047660G, rs17047661G and rs6691117G SNPs were more frequent in CD patients. Moreover, the same was observed for the rs17047661AG, rs17047661GG, rs6691117AG and rs6691117GG genotypes, indicating that they could be related to disease progression. A subsequent analysis investigated the association between SNPs and clinical manifestation, and demonstrated that rs6691117G was associated with the asymptomatic and digestive forms when compared to healthy individuals. At the same time, rs6691117AG and rs6691117GG were also more frequent in asymptomatic patients. The rs17047661 SNP was directly associated with the cardiac form for the G allele and the AG and GG genotypes. The same was observed for the rs17047661AG, rs17047661GG, rs6691117AG and rs6691117GG genotypes, indicating that they could be related to disease progression. The reconstructed haplotypes observed for the six SNPs investigated in the study (rs17259045, rs41274768, rs17047660, rs17047661, rs4844609, and rs6691117) were also evaluated, evidencing that the CD patients presented the *AGAGTG* haplotype more frequently than the control group, and cardiomyopathy was strongly associated with the *AGGGTG* haplotype. Those authors also performed LD and observed that the rs17259045, rs41274768, rs17047660 and rs17047661 SNPs were in LD with rs6691117, while rs17047660 was also in LD with rs17047661 in the CD patients. The identification of these SNPs with LD indicates an association characteristic of the infection pathological condition.

The lectin complement pathway has been widely explored in this field of research. A study investigated three SNPs from exon 7 of *COLEC11* (collectin) gene in a population of 108 healthy individuals and 251 CD patients and found increased frequencies in the rs7567833 AG and GG genotypes and the rs7567833G allele in CD patients in contrast to the control group. Haplotypes constructed by the rs148786016, rs7567833 and rs114716171 SNPs showed that the *GGC* variant was frequent among the CD patients, whereas the *GAC* haplotype was more frequently observed in the control group, thus suggesting a protective role against CD (18). A study conducted by Weitzel and colleagues (40) with 45 healthy individuals and 125 CD patients reported that the MBL2*B (mannose-binding lectin) mutation located in the *MBL2* gene was more frequently found in affected individuals compared with controls, and no association with the clinical manifestation was observed. In contrast, another research group found a negative association between the MBL2*C allele and disease progression in a study conducted with 196 chronic patients and 202 controls, and the aforementioned mutation was not observed in cardiac patients (38).

Complement pathway also plays an important role in infectious conditions caused by *Leishmania* parasites. A study addressing Visceral Leishmaniasis (VL) evaluated 218 patients and 215 controls and substantiated the association of two promoter variants of the *MBL2* gene – -78C/T and +4P/Q – with protection against VL, because the frequency observed was higher in the control group than in the affected individuals. Among the five most recurrent haplotypes, *LYQA* was more frequent in control groups compared with patients (36). Alonso and colleagues (39) also researched the influence of *MBL2* mutations on VL progression, including the clinical manifestation of the disease. The genotypes for *MBL2* were obtained from 76 healthy individuals and 159 VL patients, with 90 asymptomatic and 69 symptomatic patients. Those authors demonstrated that the genotypes related to increased MBL plasma levels were more frequent in VL symptomatic patients than in VL asymptomatic patients and healthy individuals. This gene was the target for Asgharzadeh and collaborators (41) as well, which found the presence of the Wild-Type (WT) MBL2*A allele more frequently on VL patients. This research was conducted with 58 VL patients and 120 controls in Iran.

Just like MBL and collectins, ficolins (FCN) are protagonists in the activation of the lectin pathway, and are considered a target in disease susceptibility studies addressing mutation. Research conducted in India with 225 healthy individuals and 218 VL patients reported a *FCN2* mutation demonstrably associated with disease progression. The +6359C>T structural variant, located in exon 8 of *FCN2* gene, led to a threonine-to-methionine substitution, and its TT genotype was more common in the VL patients. Among the haplotypes reconstructed from the three promoter SNPs and the exon 8 SNP evaluated in the aforementioned study (rs3124952, rs3124953, rs175114136, and rs17549193, respectively), rs3124953 and rs17549193 were in strong LD in the control group; however, the promoter variants were also in high LD with the exon 8 variant for both the control and patient groups. In addition, the *AAAC* haplotype was inversely correlated with the disease, because it was more recurrent on the healthy individuals compared with the VL patients (32). Genetic variants on the *FCN2* gene have also been assessed for Cutaneous Leishmaniasis (CL). Assaf and collaborators (8) found that the +6424G>T variant in exon 8 presented more homozygous patients for +6424T/T than healthy individuals. Haplotypes combined for the promoter SNPs (-986G>A, -602G>A, and -4A>G) and exon 8 SNPs (+6359C>T and +6424G>T) demonstrated that the *AGACG* variant was observed more frequently in the CL patients than in the controls. These promoter SNPs were also investigated by Luz and colleagues (20) regarding their influence of CD progression. In a study conducted with 305 controls and 243 patients, those authors demonstrated that the -4A/G variant was found more frequently in the healthy control group than in the cardiac group. Moreover, for the rs3124952, rs3124953, rs175114136 and rs7851696 SNPs, the *AGGA*/*GGAS* haplotypes were more frequent in cardiodigestive patients than in healthy individuals.

Only one of the 12 selected papers performed experiments with alternative pathway genes. A study conducted with 100 healthy individuals and 100 CD patients found a negative association with the *BF S* Factor B allotype, which was proved to be more frequent in control patients than in CD patients (37). The same study showed that the *C3 F* allotype was more frequent in cardiomyopathic patients compared with indetermined patients and healthy controls.

#### 3.2.2 Studies Addressing Expression Levels of Complement Genes

ELISA-based experiments performed in 221 CD patients and 102 controls for CR1 (22) demonstrated that the plasma levels of this protein were reduced in affected patients for all clinical manifestations compared with the control group. The same research group found similar results for collectin-11 in a study conducted with 233 patients and 102 controls (18). Interestingly, ficolin-2 levels were lower in CD patients than in the controls, and this was also correlated with disease severity (20). Mishra and collaborators (32) observed a direct increase in ficolin-2 serum levels in Indian VL caused by the +6359C>T structural variant. *FCN2* variants located in the promoter and exon 8 SNPs (-602G>A and +6424G>T, respectively) were associated with differences in ficolin-2 plasma levels, in which homozygotes caused higher concentrations while heterozygotes caused intermediate concentrations (8).

Mishra and colleagues (36) reported increased MBL levels in VL patients, corroborating the findings by Alonso and collaborators (39), who observed an association with disease manifestation. These studies demonstrated that MBL levels were significantly higher in symptomatic patients than in asymptomatic patients and healthy individuals.

Similar results were obtained by Boldt and collaborators (25), who found an association between low MASP-2 levels from the lectin pathway and CD patients in a study conducted with 208 CD patients and 300 healthy individuals.

**Figure 2:**
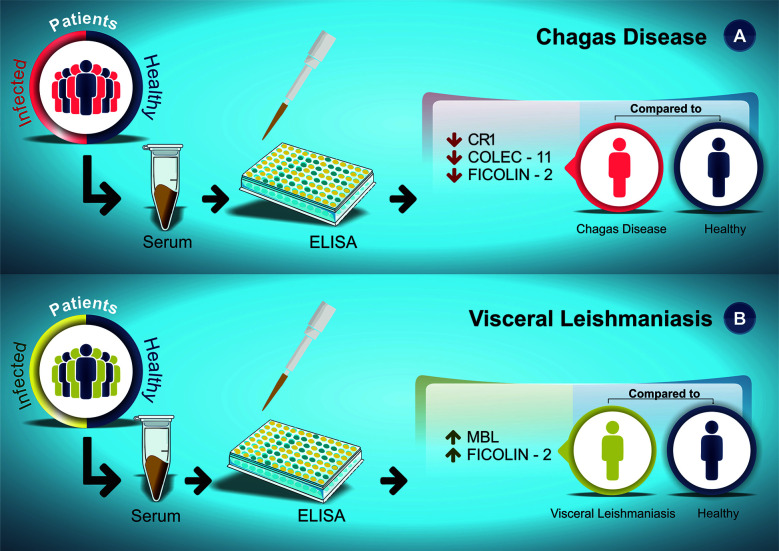
Susceptibility profile for CD **(A)** and VL **(B)** according to serum protein expression levels reported in the articles included in this systematic review. **(A)** CD patients (red) and healthy individuals (dark blue) serum levels were measured by ELISA assay. **(B)** VL patients (green) and healthy individuals (dark blue) serum levels were measured by ELISA assay. The arrows indicate the increase (↑) or decrease (↓) protein level in the serum of patients compared to the healthy control group.

Interestingly, it was evidenced how the decrease and increase in the expression levels of these proteins were directly associated with susceptibility to CD and VL, respectively, with emphasis on the ficolin-2 lectin pathway protein, which was little expressed in CD patients and highly expressed in VL patients (**Figure 2**).

Summary of gene and protein profiles associated with disease progression listed in this systematic review are given in **Table 2**.

**Table 2:** Main gene and protein alterations associated with disease progression found by articles included in this systematic review.

Reference	Gene	Target	Genetic variants associated with disease			Protein levels associated with disease
			Mutation/Haplotype*	NCBI rsSNP	Amino acid change	
Mishra et al., 2015 (32)	*FCN2*	Exon 8	+6359C>T +6359TT	rs17549193T rs17549193T	T236M −	High
Mishra et al., 2015 (36)	*MBL2*	Promoter	−	−	−	High
			−	−	−
Sandri et al., 2019 (18)	*COLEC11*	Exon 7	+39617A>G	rs7567833G	−	Low
			+39617AG	rs7567833AG		
			+39617GG	rs7567833GG	−	
			GGCG	−	−	
Assaf et al., 2012 (8)	*FCN2*	Promoter, Exon 1-8	*AGACG*	−	−	−
		Exon 8	−	−	−
Messias-Reason et al., 2003 (37)	*C3; BF*	Exon 3	+40495G>A,C,T (C3 F)	rs2230199	A80G	−
Boldt et al., 2011 (25)	*MASP2*	Promoter, Exon 3, Intron 9, Exon 10	*CDPCYV*	−	−	High
Luz et al., 2016 (38)	*MBL2*	Exon 1	−	−	−	−
Sandri et al., 2018 (21)	*CR1*	Exon 29	+113244A>G	rs17047660G		Low
			+113277A>G	rs17047661G	−	
			+113319A>G	rs6691117G		
			+113277AG	rs17047661AG		
			+113277GG	rs17047661GG		
			+113319AG	rs6691117AG		
			+113319GG	rs6691117GG	−	
			*AGAGTG; AGGGTG*	−	−	
Luz et al., 2013 (20)	*FCN2*	Promoter,	-4A>G	rs17514136G	−	Low
		Éxon 8	+6424G>T	rs7851696T	A258S
Alonso et al., 2007 (39)	*MBL2*	Promoter, Exon 1	−	−	−	High
Weitzel et al., 2012 (40)	*MBL2*	Exon 1	+3339C>T (MBL2*B)	rs1800450	G54D	−
Asgharzadeh et al., 2007 (41)	*MBL2*	Exon 1	MBL2*A (WT)	−	−	−

^*^WT, Wild-Type.

## 4 Discussion

The complement system participates actively in first-line immune defense against pathogens (43). Proteins act on pathogen opsonization in a cascade reaction and induce inflammation, which activates immune cells against the invading agent and assist in maintaining host homeostasis (44). Studies have confirmed that genetic mutations in complement genes, as well as modifications in their expression levels, are associated with development of several human disorders, such as schizophrenia (45), age-related macular degeneration (46), Alzheimer’s disease (47), myocardial infarction (48), and stroke (49). In addition, these events were also interrelated with susceptibility to several bacterial and viral infectious diseases, such as meningitis (50, 51), leprosy (52), hepatitis B (53), and diseases caused by trypanosomatids: CD (22), VL (36), and CL (8).

In most cases, the expression levels of these proteins are directly affected by mutations in their respective genes, as a cause-and-effect relationship (54); therefore, nine of the 12 selected papers included both gene and protein conditions. Exons are composed of the coding region as well as of the 3’ and 5’ untranslated regions of the RNA, thus they were considered the main targets of almost all papers. However, previous studies have elucidated how genetic alterations in the promoter region are capable of impacting on protein concentration (30, 55), activity (56), and transcriptional efficiency (57) in the organism, and nine of the selected papers also included an analysis of this regulatory region.

The lectin complement pathway is, by far, the most explored in the context of progression of infectious diseases. This is due to the fact this pathway is activated by PRMs, providing first-line immunological defense against pathogens (58) and contributing to clearance of microbes (59). Moreover, it has already been described that several protozoan species are able to activate this pathway with high efficiency, since depletion of MBL and ficolins in serum reduced the complement deposition and lysis by approximately 70% in *T. cruzi* (60). This explains why ten of the 12 selected papers included in this systematic review focused on this complement pathway.

Population genetics studies demand the inclusion of several methodological criteria to ensure their reliability. One of the fundamental principles is the assessment of HWE, which is used to estimate the number of homozygous and heterozygous variant carriers based on their allele frequency in populations that are not evolving (61). Fortunately, almost all included papers used this criterion in their methodology, which sustained the findings of association of SNPs with disease progression (22). Interestingly, while some studies analyzed the SNPs out of HWE (22, 40), others chose to remove them from the analysis (32, 36). This methodological divergence could influence the conclusion from the data obtained; therefore, it is important to adopt a standard protocol to ensure the quality of the presented evidence in future studies.

In the genetic-based context, the study of SNPs enables the formation of haplotypes, which are sets of SNPs found in the same chromosome (42). The distance between the SNPs in a haplotype allows the LD analysis, which is also important in this field since it can be associated with disease progression among its multiple applications (62). In this context, the lectin pathway, once again, plays an important role, since *FCN2* haplotypes have been associated with protection against VL (32) and susceptibility to CL (8); the *MBL2* haplotype was interrelated with protection against VL (36); the *COLEC11* haplotype was associated with susceptibility to CD (18), and the *MASP2* haplotype was related to both resistance and susceptibility to CD (25), sustaining the importance of performing this analysis.

ELISA has been the most used technique in the analysis of expression levels of complement proteins (22, 36, 38). Since this tool is antibody-based, it provides high accuracy for identification of proteins and peptides (63). However, this assay presents limitation in large-scale studies, as most ELISA kits are antigen-specific (64). Currently, there are tools that can optimize this analysis and increase the number of targets and samples, such as mass spectrometry-based proteomics (65). It enables large-scale quantitative analysis of these serum proteins by using a small sample volume, and generates considerable information regarding patient physiology; however, it should be considered that the large abundance of other proteins and their interactions could be a challenge for this strategy (66). In contrast, several techniques have been used to perform identification of SNPs, such as conventional PCR (25), real-time PCR (8), and SNaPshot Multiplex kit (39). However, most of the studies used the Sanger sequencing method (18, 32, 36), which was the first approach to be developed (67). Although this method is still used to validate more modern techniques (68), it has a high cost per run, which hinders the investigation of many targets at once (69). New Generation Sequencing (NGS) could strengthen these studies, considering that it is able to generate a large amount of data faster and cheaper (67), which broadens the research field since it allows the investigation of more genes in the same study.

Another important aspect in the context of methodology planning in a research proposal is sample size determination, which was considered only by three papers in this systematic review. It can be calculated in several ways (70) and sustains the representativity of the sample with the original population, since a small number could not generate statistical significance, and a large number could be a waste of resources (71). However, it could be unapproachable, especially in developing countries, considering that it requires funding to include several places in the study area, and there is a risk of not reaching the calculated number due to the lack of interest or availability of volunteers (72). The inclusion of diversity in experimental groups is also of vital importance, and was considered in nine of the papers. It reduces potential bias, since gender and ethnic origin directly impact on the results of a research in its various topics, including pain response (73) and autoimmune (74) and infectious diseases (75). One paper did not describe any information regarding the experimental group characteristics; therefore, it is not possible to analyze these results for bias (41). The definition of the correct control groups also sustains the truthfulness of the research results (76), and should be described clearly in the methodology section. In this context, three papers did not specify the origin of the control group (18, 20, 38), which could not represent the experimental group location if collected in a different place.

Chagas Disease was more explored than Leishmaniasis in the specific literature. The most used diagnostic tool for CD among the selected papers was based on serological methods such as immunofluorescence and antigen detection (18, 40). For VL, detection of the rk39 parasite antigen was the most used technique (32, 36), while Giemsa staining of lesion samples was the most used for CL diagnosis (8). Interestingly, none of the papers chose molecular tests, which are more accurate and can identify co-infections and recognize low antibody producing patients (77). However, some studies suggested a combination of serological and molecular assays to maximize the chances of performing a correct diagnosis (77, 78). These tests have different specificity and sensitivity rates, which should be considered in the interpretation of results (77). *T. cruzi* parasites can be classified into six different types due to propagation of polymorphism events; therefore, the targets for molecular tests should be considered carefully to include as many variants as possible (79). The correct treatment for VL depends on accurate and early diagnosis. Although the rk39 antigen-based test provides a quick and selective result, it is not able to differentiate precisely between an active and a past diagnosis. Therefore, molecular methods such as PCR should be used in parallel to ensure the correct diagnosis and monitor the treatment efficacy (80). Giemsa staining of skin lesion is widely used in CL diagnosis; however, it is not the most sensitive technique and demands experienced professionals to be performed safely. PCR is the most sensitive method, providing fast results and offering additional analysis of species identification (81). The choice of the diagnostic method is associated with the selection of the appropriate sample for the test. In this context, understanding the infection dynamics of each parasite is vital, since molecular tests detect the parasite genetic material, which is found more abundantly in their target organ. Fortunately, nearly all papers included in this systematic review were careful about this matter.

## 5 Conclusion

The complement system plays an important role in the initial phase of infectious processes, and it was proved that genetic alteration in its genes considerably affects the progression of several conditions, including diseases caused by trypanosomatids. The lectin pathway is the most explored topic of complement in the literature, and its protein levels are associated with both susceptibility and resistance to diseases.

Chagas Disease is more studied than Leishmaniasis in the specific literature, and most of the papers assessed were careful about experimental planning. However, current studies should focus on including more accurate and modern diagnosis and analysis techniques, since several complement proteins have not been investigated in this field of research yet.

## Data Availability Statement

The original contributions presented in the study are included in the article/supplementary material. Further inquiries can be directed to the corresponding author.

## Author Contributions

TT, LM, PS and FF: Study conception and design. TT and LM: Data collection. TT, LM, and PS: Data analysis. TT, LM, and PS: Data interpretation. TT and LM: Drafted the manuscript. PS and FF: Revised the manuscript. PS and FF supervised the study. All authors contributed to the article and approved the submitted version.

## Funding

This study was supported by Fundação Oswaldo Cruz (FIOCRUZ) and Conselho Nacional de Pesquisa e Desenvolvimento (CNPq) (PROEP) (442055/2019-6). FF holds a grant from CNPq for productivity in research (309862/2015-9).

## Conflict of Interest

The authors declare that the research was conducted in the absence of any commercial or financial relationships that could be construed as a potential conflict of interest.

## Publisher’s Note

All claims expressed in this article are solely those of the authors and do not necessarily represent those of their affiliated organizations, or those of the publisher, the editors and the reviewers. Any product that may be evaluated in this article, or claim that may be made by its manufacturer, is not guaranteed or endorsed by the publisher.
